# Spatiotemporal Transcriptome Profiling Reveals Nutrient Transport Dynamics in Rice Nodes and Roots During Reproductive Development

**DOI:** 10.3390/ijms26199357

**Published:** 2025-09-25

**Authors:** Wan-Chun Lu, Xiu-Lan Zheng, Yue-Tong Xiao, Zhan-Fei Sun, Zhong Tang, Fang-Jie Zhao, Xin-Yuan Huang

**Affiliations:** Sanya Institute of Nanjing Agricultural University, State Key Laboratory of Crop Genetics & Germplasm Enhancement and Utilization, College of Resources and Environmental Sciences, Nanjing Agricultural University, Nanjing 210095, China; 2019203060@njau.edu.cn (W.-C.L.); 2023103105@stu.njau.edu.cn (X.-L.Z.); 2023803214@stu.njau.edu.cn (Y.-T.X.); 2023803165@stu.njau.edu.cn (Z.-F.S.); tangzhong@njau.edu.cn (Z.T.); fangjie.zhao@njau.edu.cn (F.-J.Z.)

**Keywords:** grain filling, node, nutrient transport, mineral nutrient allocation, transcriptome profiling

## Abstract

Efficient allocation of mineral nutrients and photoassimilates is essential for grain development in rice. However, the transcriptional programs governing nutrient transport at key reproductive stages remain largely unresolved. Here, we performed a comprehensive transcriptome analysis of rice (*Oryza sativa* L.) across spatial (nodes, roots, and five other tissues) and temporal (seven reproductive stages) dimensions to elucidate the molecular basis of nutrient transport and allocation. RNA-seq profiling of node I identified stage-specific gene expression patterns, with the grain filling stage marked by strong induction of transporters involved in mineral allocation (e.g., OsYSL2, OsZIP3, OsSULTR3;3, SPDT) and carbohydrate distribution (e.g., OsSWEET13, OsSWEET14, OsMST6). Comparative analysis with the neck-panicle node (NPN) and root revealed tissue-specific regulatory networks, including nitrate (OsNRT1.1A, OsNRT2.3) and phosphate (OsPHT1;4, OsPHO1;3) transporters enriched at the grain filling stage. Root expression of Cd/As-related transporters (OsNRAMP5, OsCd1, OsLsi1, OsLsi2, OsLsi3) during grain filling highlights the contribution of belowground uptake to grain metal accumulation. Together, our study establishes a spatiotemporal atlas of nutrient transporter gene activity during rice reproductive development and identifies candidate genes regulating upward and lateral nutrient allocation. These findings provide insights into improving nutrient use efficiency and reducing toxic metal accumulation in rice grains through targeted manipulation of nodal and root transport systems.

## 1. Introduction

Efficient nutrient uptake, translocation, and allocation are essential for plant growth and development. This is particularly important during the reproductive development stage, when a large amount of mineral nutrients and photoassimilates must be mobilized from source organs such as roots and leaves to rapidly developing sink organs including the panicle and grain. Efficient long-distance transport and precise allocation of both mineral nutrients and photoassimilates are essential for sustaining reproductive development and grain filling in cereal crops such as rice (*Oryza sativa* L.). During reproductive growth stage, mineral elements including nitrogen (N), phosphorus (P), and potassium (K), and micronutrients such as iron (Fe) and zinc (Zn), as well as carbohydrates produced via photosynthesis, must be translocated from source organs to sink tissues. In this systemic nutrient flow, nodes function as critical regulatory hubs that govern the direction, timing, and magnitude of translocation between organs [1,2]. Anatomically, rice nodes are composed of a complex vascular system that includes enlarged vascular bundles (EVBs), diffuse vascular bundles (DVBs), and transit vascular bundles (TVBs), which interconnect via nodal vascular anastomoses (NVAs), enabling intervascular exchange and rerouting of transport streams [3]. These structures not only facilitate mineral nutrient redistribution to developing tissues but also mediate the unloading and redirection of photoassimilates toward the reproductive sinks, particularly during panicle exertion and grain filling. In addition to these functions, nodes also act as selective barriers or gateways that regulate the movement of toxic heavy metals such as cadmium (Cd) and arsenic (As) toward the grains. Thus, nodes serve as dynamic gateways that integrate vascular architecture with developmental and metabolic signals, enabling coordinated delivery of both inorganic and organic nutrients to support reproductive success [1,2]. Despite the central physiological roles, the dynamic gene expression pattern and molecular regulatory networks that coordinate nutrient and assimilate flow in nodes remain largely unexplored.

Recent studies have uncovered numerous transporters and regulatory genes that are specifically expressed in nodes and contribute to nutrient allocation in rice. In rice, nodes play a vital role in the allocation of macronutrients such as N, P, and K, which are required in large quantities during reproductive growth. A key player in phosphorus partitioning is the *SPDT* transporter (SULTR-like Phosphorus Distribution Transporter), which is specifically expressed in both enlarged and diffuse vascular bundles of the node. SPDT mediates the unloading of phosphate from xylem into the panicle-directed phloem; knockout of *SPDT* leads to a substantial reduction in grain phosphorus concentration and a corresponding increase in phosphorus retention in vegetative tissues, without significantly affecting grain yield [4]. For nitrogen, the vacuolar peptide transporter *OsNPF7.3* is strongly expressed in stem and node tissues under organic nitrogen supply. Overexpression of *OsNPF7.3* enhances the remobilization of nitrogen from vegetative organs to grains, resulting in increased grain nitrogen content and improved yield [5]. Regarding potassium partitioning, the phloem-localized transporter *OsHAK18* is prominently expressed in vascular tissues of rice shoots, including nodes. OsHAK18 mediates K^+^ loading into phloem and facilitates source-to-sink redistribution of potassium and sucrose, contributing to enhanced grain yield and potassium utilization efficiency [6]. Silicon (Si), although not classified as essential, is actively transported in rice and contributes to stress resistance and grain development. Three node-localized Si transporters (Lsi2, Lsi3, and Lsi6) are involved in the intervascular transfer of Si, which is required for the preferential distribution of Si to the panicle [3].

Alongside macronutrient allocation, a number of transporters mediating the movement of essential or toxic elements have been identified in rice nodal tissues. These include OsFRDL1, a citrate efflux transporter responsible for Fe solubilization and delivery to the panicle [7]; OsYSL2 and OsYSL9 from the Yellow Stripe-Like family, which mediate long-distance transport of Fe and Mn via phloem loading of metal-nicotianamine complexes [8,9]; OsOPT7 and OsVIT2, which facilitate intracellular Fe chelate transport and vacuolar sequestration [10,11]; and OsNRAMP3, involved in Mn translocation between flag leaves and panicles [12]. Zinc homeostasis is regulated by several ZIP family transporters; OsZIP3 unloads Zn from xylem into diffuse vascular bundles [13], while OsZIP1 contributes to Zn efflux, and OsZIP8 mediates Zn uptake at late developmental stages [14]. Copper is transported to upper tissues via OsHMA5, a heavy-metal ATPase involved in xylem loading of Cu in node EVBs [15]. Besides essential nutrients, nodes also function as barriers regulating the movement of toxic elements such as Cd and As. Transporters implicated include OsLCT1, which likely mediates Cd efflux from parenchyma cells to restrict Cd entry into phloem [16]; OsHMA2 and OsZIP7, involved in loading Zn and Cd into phloem [17,18]; OsZIP2, involved in root-to-shoot translocation and intravascular transfer of Cd in the nodes [19]; OsCCX2, a cation/Ca^2+^ exchanger also likely contributing to Cd intervascular transfer [20]; and the silicon transporter OsLsi2, expressed at phloem of EVBs and controlling As distribution between flag leaf and panicle [3].

Beyond mineral nutrients, nodes also contribute significantly to the redistribution of photoassimilates, primarily sucrose, during reproductive development. Key sucrose transporters such as *OsSUT1* and *OsSWEET11* are expressed in phloem-related tissues of stems and nodes, facilitating the loading and unloading of sucrose between vascular compartments. OsSUT1 mediates retrieval of apoplastic sucrose during phloem transport and ensures efficient source-to-sink connectivity [21], while OsSUT2 is involved in sucrose transport across the tonoplast from the vacuole lumen to the cytosol and plays an essential role in sugar export from the source leaves to sink organs [22]. Meanwhile, OsSWEET11, induced by the transcription factor OsDOF11, which is expressed in node-adjacent vascular tissues, is localized in companion cell–sieve element complexes of vascular bundles, promoting sucrose efflux into the phloem sap destined for developing grains [23]. In cooperation with OsSWEET11, OsSWEET15, localized in parenchymatic cells of the vascular bundle, the nucellar projection, the nucellar epidermis, and the endosperm, mediates sucrose export from vascular parenchyma into the apoplasmic space, followed by import into the endosperm [24]. These node-associated sucrose transporters underscore that nodes are active sites of carbohydrate redistribution, integrating source strength and sink demand through regulated sucrose transport mechanisms.

In cereal crops such as rice, vascular nodes act as central regulatory hubs that coordinate the long-distance translocation and targeted redistribution of mineral nutrients and photoassimilates to sink tissues such as developing grains. Simultaneously, root nutrient uptake is crucial during grain filling to maintain supply for reproductive success and yield. While many individual transporters in nodes have been studied, a comprehensive understanding of gene expression dynamics across developmental stages and tissues, along with regulatory networks controlling nutrient and assimilate flow, remains lacking. To address this, we conducted a comprehensive transcriptomic analysis focusing on key spatial and temporal dimensions during rice reproductive development. We aimed to (1) investigate the temporal dynamics of gene expression in node I, a tissue essential for allocating mineral nutrients and photoassimilates to the panicle; (2) identify node I-specific gene expression patterns by comparing transcriptomes across seven representative tissues at the grain filling stage; (3) compare transcriptional differences in the neck-panicle node (a tissue not directly involved in nutrient allocation but supporting nutrient flow to the panicle) between the early inflorescence stage and grain filling stage to dissect the functions of neck-panicle node in nutrient transport; and (4) examine differential gene expression in roots between the early inflorescence stage and grain filling stage to reveal root-level transcriptional adjustments associated with mineral uptake. These datasets provide a global view of dynamic gene expression networks in rice nodes and offer new insights into how nodes coordinate nutrient flow to support grain development and may serve as valuable resources for improving nutrient-use efficiency and grain quality through molecular breeding.

## 2. Results

### 2.1. Transcriptome Sequencing Data Overview

**Figure 1:**
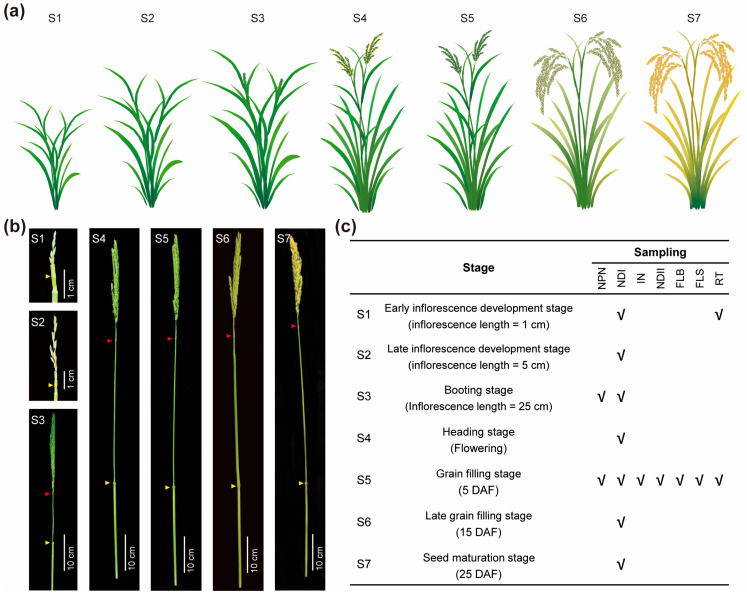
The sampling diagram during the reproductive growth stage. (**a**) Schematic diagrams of rice plants at seven different sampling stages. (**b**) Images of panicles at seven different sampling stages. The yellow arrows indicate node I; the red arrows indicate the neck-panicle nodes that are sampled at the booting stage and grain filling stage. (**c**) Detailed information of seven different sampling stages. The check marks indicate the tissues that were sampled for RNA-seq at each corresponding stage. NPN, neck-panicle node; NDI, node I; IN, internode I; NDII, node II; FLB, flag leaf blade; FLS, flag leaf sheath; RT, root; DAF, day after flowering.

To capture the temporal gene expression pattern in node I, we sampled and performed RNA-seq on node I at seven key developmental stages (Figure 1a–c): the early inflorescence development stage (S1, ~1 cm inflorescence), late inflorescence development stage (S2, ~5 cm), booting stage (S3, ~25 cm), heading stage (S4, flowering), grain filling stage (S5, 5 days after flowering), late grain filling stage (S6, 15 DAF), and seed maturation stage (S7, 25 DAF). S1–S4 correspond to the inflorescence formation phase, while S5–S7 encompass the grain filling and maturation process. Notably, stage S5 marks the transition from flowering to active grain filling, a period characterized by rapid nutrient transport and assimilate redistribution. To investigate node I-specific transcriptional signatures at the S5 stage, we collected node I (NDI) and six other tissues, including the neck-panicle node (NPN), internode I (IN), node II (NDII), flag leaf blade (FLB), flag leaf sheath (FLS), and root (RT). To further probe spatial-functional divergence between nodes, we compared NPN and NDI at both S3 and S5. Additionally, to investigate dynamic shifts in nutrient acquisition capacity, root transcriptomes were analyzed at both S1 and S5 (Figure 1).

All samples were collected in three biological replicates from different individual plants, yielding a total of 45 samples representing 15 distinct tissue–stage combinations (Table S1). RNA-seq generated over 12 billion raw reads in total. After quality filtering, each library retained an average of ~25.37 million clean reads. Quality assessment revealed Q30 values exceeding 93%, and an average GC content of ~52% across all libraries. Approximately 95% of clean reads were successfully aligned to the Nipponbare reference genome (IRGSP-1.0), with over 90% of reads uniquely mapped and less than 4% classified as multi-mapped and excluded from downstream analyses (Table S1).

### 2.2. Gene Expression Pattern of Node I During the Reproductive Growth Stage

**Figure 2:**
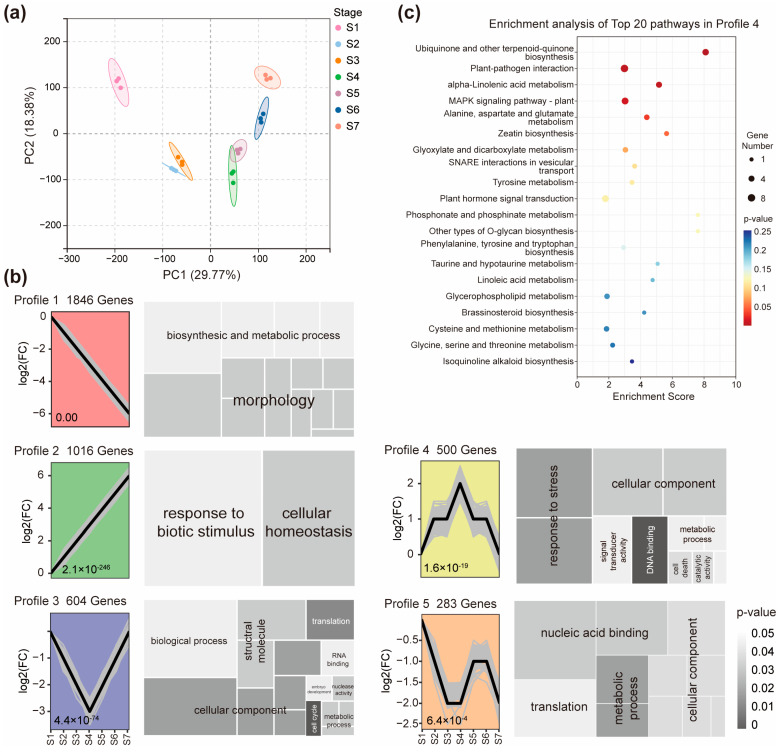
Dynamic gene expression pattern in node I during the reproductive growth stage. (**a**) Principal component analysis (PCA) of gene expression profiles in node I at seven reproductive growth stages. Each data point represented an independent biological replicate. (**b**) Short Time-series Expression Miner (STEM) analysis of genes in node I at seven reproductive growth stages. Five significant profiles with color background are shown. *p*-values were represented below each profile, and gene numbers in each profile are shown on the top. The tree map on the right side of each profile is GO enrichment analysis. The functional categories with false discovery rates ≤ 0.05% were summarized using REVIGO. The grey color indicates the significance of the GO terms, and the aggregate size indicates the gene numbers of the GO terms. (**c**) KEGG pathway enrichment analysis of genes in profile 4. The top 20 KEGG pathways with the lowest *p*-values are shown.

We conducted RNA-seq on NDI samples collected at seven pivotal reproductive stages (S1–S7) covering the entire developmental timeline from early inflorescence initiation through panicle formation to grain filling and maturation (Figure 1). These stages include inflorescence initiation (S1), early inflorescence development (S2–S3), panicle morphogenesis (S4), and the grain filling to maturation phases (S5–S7), enabling comprehensive analysis of transcriptomic dynamics during reproductive growth. Principal component analysis (PCA) of gene expression profiles revealed clear temporal separation among samples (Figure 2a). The main principal component (PC1) explained 29.77% of the variance, while the second principal component (PC2) explained 17.19% of the variance. Three biological replicates of each tissue were generally grouped together, suggesting that the transcriptome data were reliable, with high repeatability (Figure 2a). Notably, S1 was clustered distinctly from later stages, reflecting the shift from inflorescence initiation to panicle formation. In contrast, S2 and S3 grouped more closely, as did S4 and S5 (Figure 2a), suggesting relatively similar gene expression programs between these adjacent stages. A major transcriptomic transition was observed at S4, coinciding with the onset of grain filling and the associated reprogramming of nutrient allocation processes. This pattern indicates that transcriptional changes in node I occur in a gradual and stage-coordinated manner, rather than through abrupt switches.

To further explore temporal expression patterns, we performed Short Time-series Expression Miner (STEM) analysis, which grouped all expressed genes in node I into 20 model profiles (Figure S1). Five of these profiles (Profile 1–5) were significantly enriched (*p* < 0.05), representing dominant transcriptional trends in node I during reproductive development (Figure 2b). For instance, Profile 1 contained 1846 genes whose expression decreased progressively across the reproductive stages (Figure 2b, Table S2). GO analysis indicated enrichment in biosynthetic and metabolic processes, including cell wall organization and developmental morphology. Key KEGG pathways included motor protein regulation, oxidative phosphorylation, and amino sugar metabolism, reflecting a downregulation of structural and metabolic activity as reproductive tissues mature (Figure S2a). Profile 2 included 1016 genes that were steadily upregulated from S1 to S7 (Figure 2b, Table S3). Enriched GO terms included “response to biotic stimulus” and “cellular homeostasis,” suggesting gradual enhancement of defense and stress adaptation pathways. The most significantly enriched KEGG pathway in this profile was glycerophospholipid metabolism, potentially reflecting changes in membrane remodeling and signaling (Figure S2b).

Profile 3 contained 604 genes that were initially downregulated, reached their lowest expression at S4, and then gradually recovered during grain filling (Figure 2b, Table S4). GO terms included “biological process,” “structural molecule activity,” and “cellular component,” while KEGG analysis highlighted ribosome biogenesis, nucleocytoplasmic transport, and RNA degradation, consistent with dynamic regulation of translation machinery and cellular architecture (Figure S2c). Profile 4 included 500 genes with expression peaking at S4, coinciding with flowering (Figure 2b, Table S5). These genes were enriched in stress response pathways and DNA-binding regulatory functions, as well as signal transduction components. KEGG analysis showed significant enrichment in ubiquinone and terpenoid-quinone biosynthesis, MAPK signaling, and SNARE-mediated vesicle transport (Figure 2c), indicating activation of redox and signaling networks at the onset of grain filling. Profile 5 contained 283 genes with more complex or less defined expression trends (Figure 2b, Table S6). Despite this variability, enriched functional categories included “nucleic acid binding,” “translation,” and “metabolic process.” The base excision repair pathway was the most significantly enriched in KEGG, suggesting roles in genome stability and transcriptional regulation under changing developmental contexts (Figure S2d).

Collectively, these time-resolved expression profiles demonstrate that node I undergoes continuous and coordinated transcriptional reprogramming throughout reproductive development. The S4–S5 transition, in particular, marks a major regulatory inflection point, coinciding with the onset of grain filling and the upregulation of genes involved in transport, stress adaptation, and metabolic redistribution.

### 2.3. Genes with a Stage-Specific Expression Pattern in Node I Across Reproductive Growth

**Figure 3:**
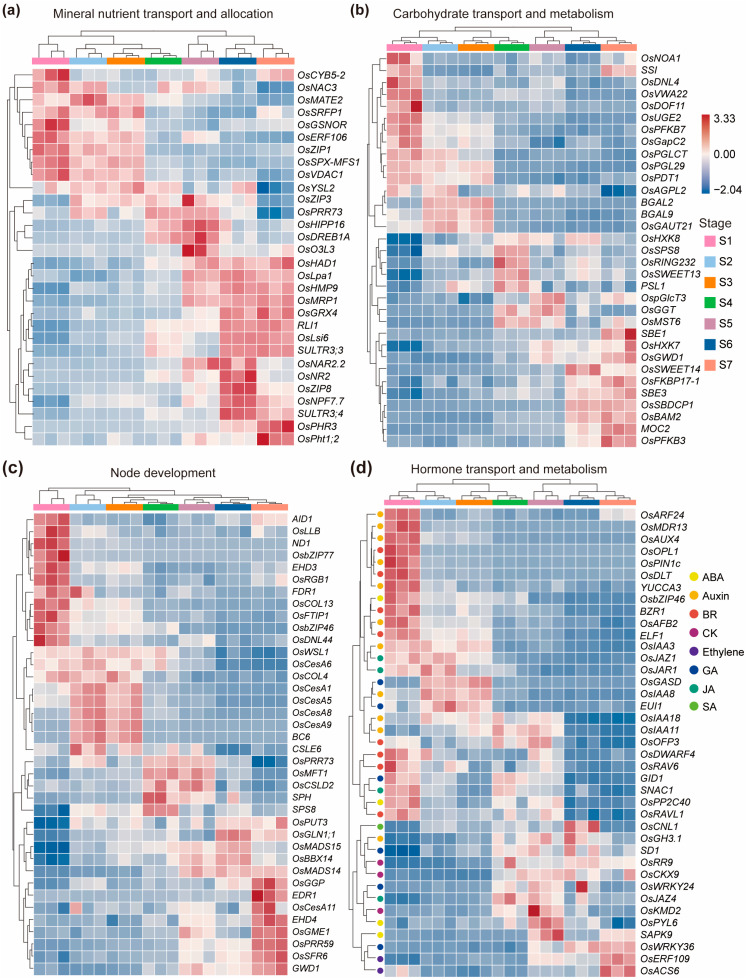
Representative genes with stage-specific expression pattern in node I during reproductive growth stage. Genes differentially expressed in particular stages related to mineral nutrient transport and allocation (**a**), carbohydrate transport and metabolism (**b**), node development (**c**), and hormone transport and metabolism (**d**) are shown. Gene expression levels are normalized to Z-scores and clustered by the average linkage method. S1 to S7 represent seven different growth stages during the reproductive growth stage. The red color in the heatmaps represents higher expression levels, while blue represents lower expression levels. The color dots beside genes in (**d**) indicate genes related to different hormones. ABA, abscisic acid; BR, brassinosteroids; CK, cytokinin; GA, gibberellin; JA, jasmonic acid; SA, salicylic acid.

To identify genes associated with dynamic physiological roles of node I, we examined representative genes with distinct temporal expression patterns across reproductive development. We focused on genes that were involved in four functional categories, including mineral nutrient transport and allocation, carbohydrate transport and metabolism, node development, and hormone biosynthesis and metabolism (Figure 3a–d, Table S7). A subset of mineral transporter genes exhibited high expression during early reproductive stages (S1–S4) and decreased thereafter (Figure 3a, Table S7). For instance, *OsYSL2*, which encodes a transporter of Fe^2+^- and Mn^2+^-nicotianamine (NA) complexes mediating long-distance translocation of Fe and Mn from vegetative tissues into grains [8], was strongly expressed during early stages, particularly in developing reproductive tissues. Members of the ZIP (ZRT/IRT-like Protein) family, including *OsZIP1*, *OsZIP3*, and *OsZIP8*, also showed relatively high expression in node I during reproductive stages. These genes are involved in the transport of divalent cations such as Zn^2+^ and Fe^2+^ and are thought to contribute to micronutrient homeostasis and intervascular redistribution [13,25,26]. Most of the nutrient transporter genes displayed peak expression in node I at the grain filling stage (S5–S6). For example, *OsLsi6*, a node-specific silicon transporter [3], showed its highest transcript levels at S5–S6. Likewise, *OsSULTR3;3* [27] and *SPDT/OsSULTR3;4* [4], encoding sulfur transporter family members with phosphate transport activity, were also upregulated during S5–S6. These transporters function in the radial movement of phosphate within the nodal vasculature, coordinating the redistribution of phosphorus from source tissues to sink organs during grain development.

In parallel with mineral nutrients, the efficient allocation of carbohydrate assimilates, particularly sucrose and its downstream metabolites, is critical for sustaining grain development during reproductive growth. Following the peak in mineral transporter activity at S5–S6, a number of genes involved in carbohydrate transport and metabolism were also found to be expressed in node I at particular stages during reproductive growth (Figure 3b, Table S7). *OsSWEET13* [28] and *OsSWEET14* [29] are two key sucrose transporters that were both upregulated in node I during the grain filling stage. In addition to disaccharide transport, monosaccharide transporters are involved in the fine-tuning of sugar flow between different cell layers and compartments within the node. *OsMST6*, a member of the monosaccharide transporter (MST) family [30], exhibited increasing expression from booting to grain filling stages, suggesting a role in hexose import or redistribution across nodal tissues. Similarly, *OspGlcT3*, a putative plastidic glucose transporter [31], was also upregulated during grain filling and may function in intercellular hexose transfer to support local energy supply or biosynthetic processes in nodal cells. Beyond sugar transporters, genes involved in carbohydrate modification and sugar metabolism also showed significant transcriptional responses in node I during reproductive development, including the galacturonosyl-transferase gene *OsPDT1* [32] and the cytosolic fructose-1,6-bisphosphatase gene *MOC2* [33].

The structural and functional integrity of node I is essential for maintaining vascular connectivity and sustaining efficient nutrient and assimilate transport during reproductive development. As the developmental and physiological context of the node changes, a coordinated program of cell wall biosynthesis and transcriptional regulation supports tissue expansion, vascular differentiation, and eventual maturation. Our transcriptome data revealed dynamic and stage-specific expression of genes involved in cell wall synthesis and developmental control, suggesting their distinct roles at different reproductive stages (Figure 3c, Table S7). Several cellulose synthase and cellulose synthase-like genes exhibited high expression during the early to mid-stages of reproductive development. Notably, *ND1*, which encodes a cellulose synthase-like protein D4 [34], showed exclusive high expression at S1, indicating its specific role during early node initiation and primary wall formation. A group of classical cellulose synthase genes, including *OsCesA1*, *OsCesA5*, *OsCesA6*, *OsCesA8*, *OsCesA9*, *BC6* (a cellulose synthase A catalytic subunit), and *CSLE6* (a cellulose synthase-like protein E6) [35,36,37], displayed peak expression at S2 and S3 and declined sharply after S4. Interestingly, *OsCSLD2*, another member of the cellulose synthase-like family, showed a delayed expression peak at S4 and S5. In contrast, *OsCesA11* exhibited a distinct expression pattern, with transcript levels peaking only at S7. In addition to structural genes, transcription factors related to developmental regulation and environmental response also displayed temporally restricted expression in node I. *OsbZIP46* [38] and *OsbZIP77* [39], both members of the bZIP transcription factor family, were specifically expressed at S1, suggesting their potential roles in initiating developmental signaling and early vascular specification in nodal tissues. On the other hand, *OsMADS14* [40] and *OsMADS15* [41], key regulators of reproductive development and phase transition, were highly expressed during S5 to S7. Their upregulation coincided with active grain filling and may reflect transcriptional reprogramming associated with late-stage nodal differentiation and reproductive commitment.

The developmental and physiological roles of node I are tightly regulated by hormonal signaling networks that coordinate organ identity, vascular activity, and source–sink communication. Multiple genes involved in the biosynthesis, transport, and signaling of major phytohormones, including auxin, brassinosteroids (BR), gibberellin (GA), cytokinin, and ethylene, exhibited distinct stage-specific expression patterns in node I, highlighting their temporal regulatory functions during reproductive development. During the early reproductive stage (S1), node I showed a strong enrichment of genes associated with auxin and BR biosynthesis and signaling. For example, *YUCCA3*, a flavin monooxygenase involved in the indole-3-acetic acid (IAA) biosynthesis pathway [42], was specifically upregulated at S1. Concurrently, *OsAUX4* (an auxin influx transporter) and *OsMDR13* (a multidrug resistance protein mediating auxin efflux) were also highly expressed, suggesting active auxin transport across vascular tissues [43,44]. The coordinated expression of *OsPIN1c*, a canonical auxin efflux carrier [45], further supports the existence of polar auxin transport mechanisms in node I at this stage. Downstream auxin response genes, including *OsARF24* (auxin response factor) [46] and *OsAFB2* (auxin receptor) [47], were also enriched at S1, indicating that the entire auxin signaling cascade is activated during early nodal morphogenesis. In parallel, several genes related to brassinosteroid signaling were also upregulated at S1. BZR1, a central transcriptional regulator in the BR signaling cascade [48], showed high expression, suggesting BR-mediated control of cell elongation and vascular differentiation. Similarly, the GAI-RGA-SCR (GRAS) family protein *OsDLT* [49] and transcription factors *OsRAV6* [50] and *OsRAVL1* [51], both involved in BR response and homeostasis, were specifically expressed in node I at S1.

At stages S2–S3, genes associated with gibberellin metabolism were differentially expressed. Notably, *OsGASD*, a positive regulator of gibberellin biosynthesis [52], was upregulated, suggesting increased GA production during the period of vascular elongation and expansion. In parallel, *EUI1*, encoding a GA-deactivating enzyme [53], was also expressed during this stage, indicating the need for tight spatial and temporal regulation of active GA levels. The co-expression of these two genes suggests a fine-tuned GA homeostasis mechanism operating in node I to coordinate tissue growth during panicle elongation and structural maturation. As node I transitions into its transport-intensive role during early grain filling (S4–S5), genes related to cytokinin metabolism and signaling showed prominent expression. *OsCKX9*, encoding a cytokinin oxidase/dehydrogenase that degrades active cytokinins [54], was significantly upregulated, indicating a potential shift in cytokinin homeostasis during the grain loading phase. Meanwhile, *OsRR9* and *OsKMD2*, both components of the cytokinin signaling pathway [55,56], were also highly expressed. These genes likely mediate cytokinin-regulated modifications in vascular activity or nutrient partitioning, contributing to the regulation of sink strength and phloem unloading during this critical developmental window. At the final stage of reproductive development (S7), genes involved in ethylene biosynthesis and response became highly expressed in node I. *OsACS6*, which encodes ACC synthase, a key enzyme in ethylene biosynthesis [57], showed peak expression at S7. This was accompanied by strong upregulation of *OsERF109*, an ethylene response factor [58], suggesting that ethylene signaling may be involved in late-stage node remodeling or senescence.

### 2.4. Genes Specifically Upregulated at the Early Grain Filling Stage (S5) in Node I

**Figure 4:**
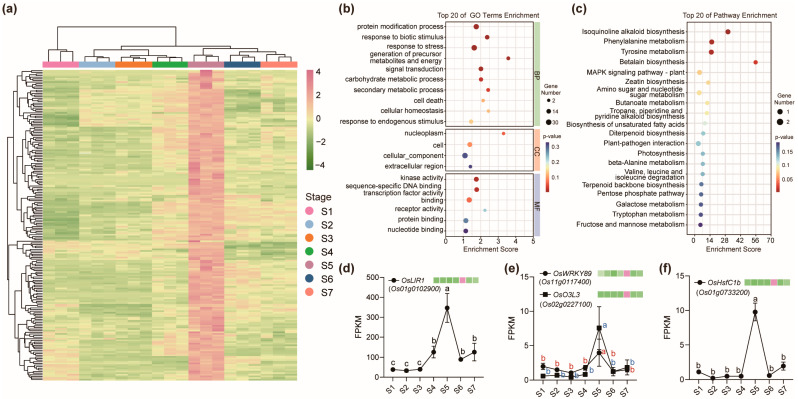
Genes specifically upregulated at the early grain filling stage (S5) in node I. (**a**) Heat map showing genes significantly upregulated at S5 in node I. The gene-normalized signal intensities are shown in the heat maps using Z-scores and clustered by the average method. S1 to S7 represent seven different growth stages during the reproductive growth stage. The red color in the heatmaps represents higher expression levels, while green represents lower expression levels. (**b**,**c**) GO enrichment analysis (**b**) and KEGG enrichment analysis (**c**) of DEGs in (**a**). The top 20 GO terms or the KEGG pathways with the lowest *p*-values are shown. (**d**–**f**) Four representative genes specifically upregulated at S5 in node I are shown. Data are shown as mean ± SD with three independent biological replicates. Different letters indicate significant differences of gene expression levels among different stages at *p* ≤ 0.05 with three biological replicates (Tukey’s multiple comparisons test). FPKM, Fragments Per Kilobase of transcript per Million mapped reads.

The grain filling stage represents a metabolically intensive period in rice reproductive development, with S5 recognized as the onset of rapid assimilate accumulation and the peak grain filling rate. To investigate the genetic basis of nodal function during this critical stage, we identified a set of 189 genes that were specifically and strongly upregulated in node I at S5, while remaining minimally expressed at other developmental stages (Figure 4a, Table S8). The expression of 11 genes that specifically expressed in node I at S5 were confirmed by qRT-PCR (Figure S3). To gain insight into the biological roles of these S5-specific genes, GO and KEGG enrichment analyses were performed. Among the top 20 significantly enriched GO terms, half were categorized under biological process, with the remainder distributed across cellular component and molecular function categories (Figure 4b). These genes were particularly enriched in processes such as protein modification, response to biotic stimulus, and kinase activity. KEGG pathway analysis revealed enrichment in isoquinoline alkaloid biosynthesis, amino acid metabolism, and betalain biosynthesis (Figure 4c), indicating that node I at S5 is transcriptionally primed to support diverse metabolic and signaling activities.

Several individual genes within this set have been functionally characterized and exhibit regulatory roles relevant to grain filling. For example, *OsLIR1* (*LIGHT-INDUCED RICE1*) was one of the most highly upregulated genes in node I at S5. *OsLIR1* encodes a chloroplast-targeted protein that interacts with LEAF-TYPE FERREDOXIN-NADP^+^ OXIDOREDUCTASE (LFNR), a key enzyme in photosynthetic electron transport, and facilitates its membrane attachment via the TIC62 anchor protein [59]. This membrane tethering enhances NADPH production efficiency, which is critical for supporting high metabolic demand during grain filling. OsLIR1 undergoes light-dependent degradation, allowing dynamic regulation of electron distribution in chloroplasts in response to environmental changes. Its peak expression at S5 suggests a role in maintaining energy balance and metabolic homeostasis in nodal vascular tissues, possibly optimizing the reductive capacity required for assimilate translocation or stress defense during rapid nutrient allocation to the grain. Another S5-upregulated gene, *OsWRKY89*, encoding a transcription factor associated with flowering time and plant height, displayed S5-specific expression in node I. While overexpression of *OsWRKY89* during early reproductive stages has been shown to delay flowering and reduce shoot elongation, its induction at S5 may instead enhance biotic and abiotic stress tolerance during grain filling, thereby safeguarding yield stability under environmental challenges [60]. *OsO3L3*, which encodes a histone H2A-interacting nuclear protein expressed in vascular tissues [61], was also highly expressed at S5. This gene has been linked to Cd partitioning in rice, with knockout lines showing increased Cd accumulation in grains. Its upregulation in node I during S5 may thus reflect a protective mechanism that restricts Cd translocation to reproductive sinks while maintaining the homeostasis of other essential metals such as Cu, Fe, Zn, and Mn. In addition, *OsHsfC1b*, encoding a heat shock transcription factor responsive to ABA [62], also exhibited S5-specific expression. Known for its role in enhancing tolerance to multiple stresses, OsHsfC1b may contribute to stabilizing the grain filling process under stress-prone field conditions by reinforcing nodal stress resilience (Figure 4d–f).

### 2.5. Spatially Distinct Gene Expression in Nodal Tissues During Rice Grain Filling

Following the identification of genes specifically upregulated at S5 in node I, we next explored whether the nodes exhibit spatially distinct transcriptional features compared to other tissues during grain filling. To investigate the tissue-specific transcriptional landscape, we conducted a comparative transcriptomic analysis between node I (NDI) or node II (NDII) and five tissues during the early grain filling stage, including neck-panicle node (NPN), internode I (IN), flag leaf blade (FLB), flag leaf sheath (FLS), and root (RT).

**Figure 5:**
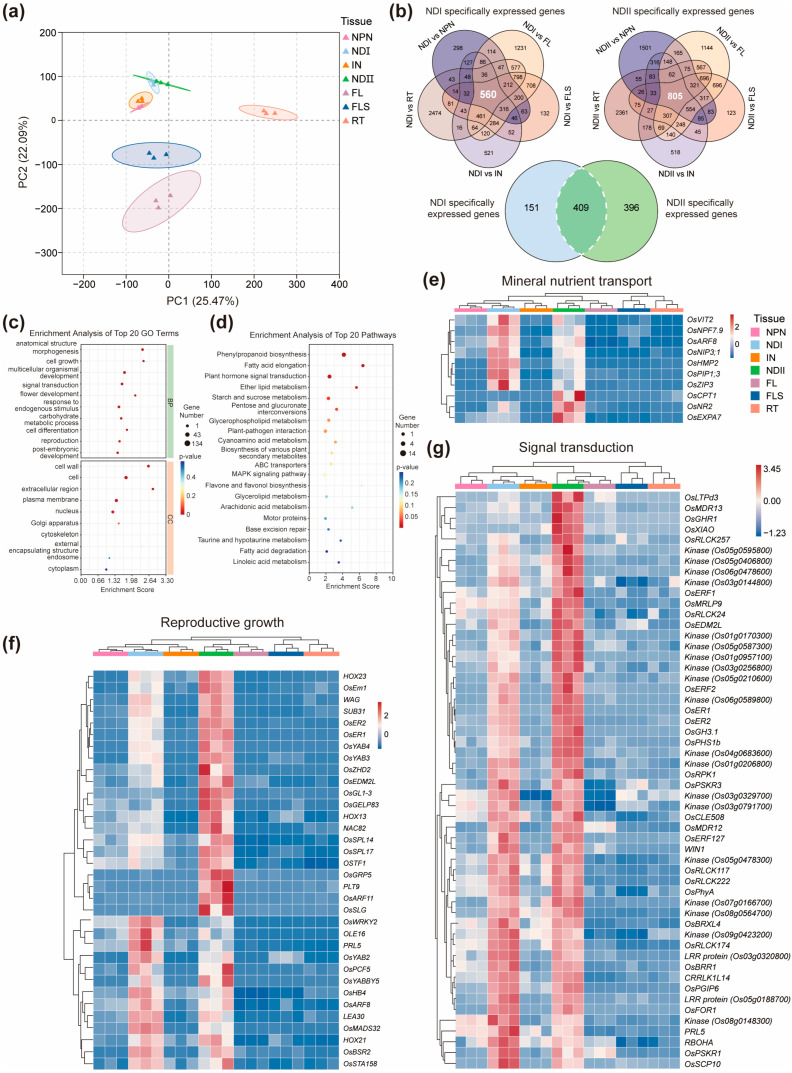
Spatially distinct gene expression in nodal tissues during the grain filling stage. (**a**) PCA analysis on seven tissues at the grain filling stage. Each data point represents an independent biological replicate. (**b**) Venn diagrams showing genes specifically expressed in node I, node II, or in both nodes. (**c**,**d**) GO enrichment analysis (**c**) and KEGG enrichment analysis (**d**) of genes specifically expressed in both node I and node II. The top 20 GO terms or KEGG pathways with the lowest *p*-values are shown. (**e**–**g**) Heat map showing genes specifically expressed in both node I and node II that were involved in mineral nutrient transpornt (**e**), reproductive growth (**f**) and signal transduction (**g**). The red color in the heatmaps in (**e**–**g**) represents higher expression levels, while blue represents lower expression levels. NPN, neck-panicle node; NDI, node I; IN, internode I; NDII, node II; FLB, flag leaf blade; FLS, flag leaf sheath; RT, root.

PCA based on FPKM values revealed clear separation among different organs, with PC1 explaining 25.47% of the variance and clearly distinguishing roots from aboveground tissues. PC2 (22.09%) further contributed to organ-specific clustering, particularly among nodes/internodes, FLS, and FLB (Figure 5a). NDI and NDII, and NPN and IN, were generally grouped together, suggesting similar transcriptomic profiling between these tissues. We then conducted a comparative transcriptomic analysis between NDI and NPN, IN, FLB, flag FLS, and RT, and identified 560 genes whose expression levels were significantly higher in NDI compared to other tissues, which were defined as specifically highly expressed in node I during the grain filling (Figure 5b, Table S9). Similar comparative transcriptomic analysis between NDII and five other tissues was conducted, and we identified 805 genes that were specifically highly expressed in node II (Figure 5b, Table S9). Venn diagram analysis revealed 409 genes that were specifically highly expressed in both node I and II (Figure 5b, Table S9). GO enrichment analysis indicated that these 409 genes distinctly expressed in nodal tissues genes were significantly associated with biological processes related to anatomical structure morphogenesis, while KEGG pathway enrichment highlighted phenylpropanoid biosynthesis, suggesting a role in structural remodeling and metabolic adaptation to support active nutrient and assimilate transport (Figure 5c–d).

Several well-characterized transport-related genes were identified among the node-specific DEGs (Figure 5e). For example, *OsVIT2*, a vacuolar membrane Fe transporter known to coordinate Fe, Zn, and Mn partitioning between source leaves and panicles [63], exhibited the highest expression in node I. Its enhanced activity in nodal vasculature likely facilitates the fine-tuned allocation of micronutrients into developing grains. Similarly, *OsZIP3*, a Zn transporter localized in xylem parenchyma and transfer cells of enlarged vascular bundles, showed node I-specific high expression. This spatial specificity is critical for restricting Cd translocation while promoting Zn accumulation in grains [13]. In contrast, *OsNR2*, a nitrate reductase gene associated with nitrogen use efficiency and yield improvement [64], showed preferential expression in node II, suggesting tissue-specific roles in nitrogen metabolism.

Several transcriptional regulators and hormonal genes involved in reproductive development also showed strong node-specific expression (Figure 5f). For example, *PRL5*, which encodes a gibberellin biosynthesis enzyme affecting panicle elongation and branching [65], was highly expressed in both node I and node II, possibly enhancing GA supply to developing panicles. *OsYAB4*, a transcription factor known to regulate vascular differentiation [66], exhibited strong expression in the nodal tissue, indicating a potential role in expanding phloem area to promote assimilate transport to the grain. Genes associated with signal transduction were also enriched in node I and node II during S5, including genes encoding protein kinases and receptor-like kinases (Figure 5g), which are known to participate in sensing developmental cues and mediating inter-organ communication.

### 2.6. Differentially Expressed Genes in the Neck-Panicle Node Between the Booting Stage and the Grain Filling Stage

**Figure 6:**
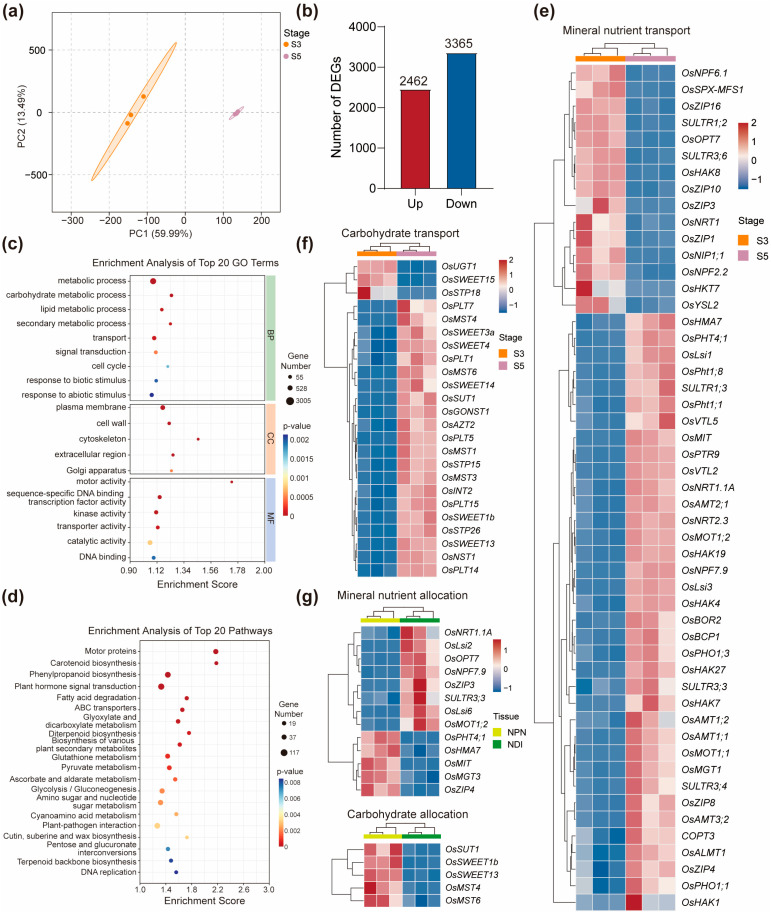
Differentially expressed genes in the neck-panicle node between the booting stage and the grain filling stage. (**a**) PCA analysis on the neck-panicle node at the booting stage (S3) and the grain filling stage (S5). Each data point represents an independent biological replicate. (**b**) Number of DEGs in the neck-panicle node between S3 and S5. (**c**,**d**) GO enrichment analysis (**c**) and KEGG enrichment analysis (**d**) of DEGs in (**b**). The top 20 GO terms or KEGG pathways with the lowest *p*-value are shown. (**e**,**f**) Heatmaps showing representative DEGs related to mineral nutrient transport (**e**) and carbohydrate transport (**f**) at S3 and S5. (**g**) Potential genes involved in mineral nutrient allocation and carbohydrate allocation in node I. Heatmaps show the normalized expression values normalized by the Z-score method. The red color in the heatmaps in (**e**–**g**) represents higher expression levels, while blue represents lower expression levels. NPN, neck-panicle node; NDI, node I.

The neck-panicle node (NPN) serves as the final hub for translocating mineral nutrients and photoassimilates into the developing panicle, playing a pivotal role in coordinating source–sink relationships in rice. To investigate transcriptional reprogramming in the NPN during the transition from booting to grain filling, we conducted RNA-seq analysis of NPN tissues sampled at the booting stage (S3) and the early grain filling stage (S5), each with three biological replicates. PCA analysis revealed clear separation of S3 and S5 samples along PC1, which accounted for 59.99% of the total variance, indicating marked transcriptomic divergence between the two stages (Figure 6a). Differential expression analysis identified a total of 5827 differentially expressed genes (DEGs) between S3 and S5, including 2462 upregulated and 3365 downregulated genes at S5 (Figure 6b, Table S10). GO and KEGG enrichment analyses revealed that these DEGs were predominantly associated with metabolic processes, transport, carotenoid biosynthesis, phenylpropanoid biosynthesis, and plant hormone signaling (Figure 6c–d), suggesting dynamic changes in cellular activities underpinning developmental and physiological shifts between the two stages.

Among these DEGs, many genes involved in mineral nutrient transport were significantly upregulated at S5 compared to S3, reflecting the increased nutrient demand and translocation during grain filling (Table S10). For example, nitrate transporter genes *OsNRT1.1A* [67] and *OsNRT2.3* [68] showed higher expression at S5, as did several ammonium transporter genes, including *OsAMT1;1*, *OsAMT1;2*, *OsAMT2;1*, and *OsAMT3;2* (Figure 6e, Table S10). Similarly, a broad set of phosphate transporter genes, such as *OsPHT1;1*, *OsPHT1;8*, *OsPHT4;1*, *OsPHO1;1*, and *OsPHO1;3*, were strongly upregulated at S5, indicating the enhanced Pi distribution during the grain filling stage. A comparable expression pattern was observed for genes related to carbohydrate transport. Five members of the SWEET family genes, including *OsSWEET1b*, *OsSWEET3a*, *OsSWEET4*, *OsSWEET13*, and *OsSWEET14*, were significantly more highly expressed at S5 than at S3 (Figure 6f, Table S10), consistent with their roles in sugar export and phloem unloading into grains. Notably, *OsSWEET15* exhibited an inverse expression trend, being more abundant at S3, possibly reflecting early-stage phloem loading or sugar storage functions [8]. In contrast to the general upregulation trend observed for nitrogen, phosphorus, and sugar transporters at S5, ZIP family transporters involved in Zn and other micronutrient transport exhibited a more complex temporal expression pattern [13,26]. For example, *OsZIP1*, *OsZIP3*, *OsZIP10*, and *OsZIP16* were more highly expressed at S3, whereas *OsZIP4* and *OsZIP8* were upregulated at S5 (Figure 6e, Table S10). These patterns suggest that different members of the *ZIP* gene family may participate in temporally coordinated phases of micronutrient mobilization to the developing panicle, reflecting stage-specific regulatory needs for grain nutrient loading.

Node I acts as a distribution center that allocates mineral nutrients transported upward from lower internodes to both the developing panicle and the flag leaf, while also directing photoassimilates synthesized in the flag leaf toward the panicle. In contrast, the neck-panicle node (NPN) is functionally unidirectional, channeling resources exclusively into the panicle. This anatomical and physiological divergence provides a basis for transcriptomic comparisons to identify genes potentially involved in the allocation of nutrients to the flag leaf. Several genes encoding mineral transporters were significantly more highly expressed in NDI than in NPN during grain filling, including the phosphate transporter gene *OsSULTR3;3*, nitrate transporter gene *OsNRT1.1A*, ZIP family transporter gene *OsZIP3*, Si transporter genes *OsLsi2* and *OsLsi6*, and molybdate transporter gene *OsMOT1;2* (Figure 6f, Table S10). The preferential expression of these genes in NDI suggests their involvement in nutrient allocation to the flag leaf. Conversely, genes related to carbohydrate transport, such as *OsSWEET1b*, *OsSWEET13*, and *OsSUT1*, showed higher expression levels in the NPN compared to NDI, indicating a dominant role in delivery of carbohydrate to the panicle during grain filling stage.

### 2.7. Differentially Expressed Genes in Roots Between the Early Inflorescence Stage and the Grain Filling Stage

**Figure 7:**
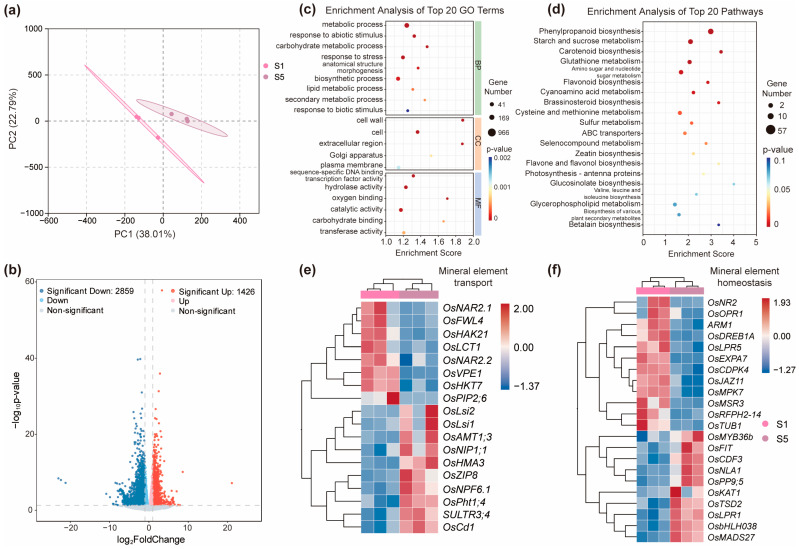
Differentially expressed genes in roots between the early inflorescence stage and the grain filling stage. (**a**) PCA analysis on the root at the early inflorescence stage (S1) and the grain filling stage (S5). Each data point represents an independent biological replicate. (**b**) Identification of DEGs expressed in root at S5 compared with S1. (**c**,**d**) GO enrichment analysis (**c**) and KEGG enrichment analysis (**d**) of DEGs in (**b**). The top 20 GO terms or KEGG pathways with the lowest *p*-values are shown. (**e**) Genes related to mineral nutrient transport that were upregulated at S5 are shown. (**f**) Differentially expressed genes related to mineral nutrient homeostasis are shown. Heatmaps in (**e**,**f**) were generated by using normalized Z-scores and clustered by the average linkage method. The red color in the heatmaps in (**e**,**f**) represents higher expression levels, while blue represents lower expression levels.

Successful grain filling in rice relies not only on the translocation of stored nutrients and photoassimilates from vegetative tissues such as leaves and stems, but also on the continuous absorption of mineral nutrients by roots during this stage. The ability of roots to meet the increasing nutrient demands of developing grains is therefore critical to reproductive success and final yield. To examine differential gene expression in roots between the early inflorescence stage (S1) and the grain filling stage (S5), and to identify root-level transcriptional adjustments associated with mineral nutrient uptake, we performed RNA-seq on root samples collected at both stages. PCA analysis revealed clear separation between the two developmental time points, with PC1 and PC2 explaining 38.01% and 22.79% of the total variance, respectively (Figure 7a). Using a fold-change cutoff of ≥ 2 and a false discovery rate (FDR) < 0.01, we identified 1426 genes significantly upregulated at S5 and 2859 genes downregulated relative to S1 (Figure 7b, Table S11). GO enrichment analysis indicated that these DEGs were mainly associated with metabolic processes and responses to stress (Figure 7c), while KEGG pathway analysis highlighted enrichment in phenylpropanoid biosynthesis and starch and sucrose metabolism pathways (Figure 7d).

Given the crucial role of roots in supplying nutrients to support grain development, we focused our analysis on genes involved in mineral nutrient transport and homeostasis at S5. Several key transporter genes showed pronounced S5-specific expression in the root (Figure 7e). For phosphorus acquisition, the high-affinity phosphate transporter *OsPHT1;4* was significantly upregulated at S5, likely contributing to sustained Pi delivery to developing grains [69]. Similarly, genes encoding nitrate transporter *OsNRT1.2* and ammonium transporters such as *OsAMT1;3* exhibited elevated expression at S5, enhancing root nitrogen uptake under strong reproductive demand. The potassium transporter *OsHAK17* and multiple silicon transporters (*OsLsi1*, *OsLsi2*, and *OsLsi3*) also showed strong upregulation at S5, indicating their roles in maintaining ionic balance and enhancing panicle rigidity and defense during filling. Furthermore, metal transporter genes such as *OsNRAMP5* and *OsCd1* (cadmium uptake), and *OsBOR1* (boron transport), were also preferentially expressed at S5, reflecting enhanced trace element acquisition during this critical developmental window. In addition to nutrient transporters, several transcriptional regulators involved in mineral nutrient signaling exhibited stage-specific expression patterns (Figure 7f). *OsMYB2P-1*, *OsMYB47*, *OsMYB67*, and *OsbZIP01* were predominantly expressed at S1, suggesting roles in early-stage root development and nutrient sensing [70,71]. In contrast, *OsMYB36b*, *OsbZIP79*, and the *bHLH* transcription factor *FIT* were upregulated at S5, implicating them in the transcriptional activation of mineral transport pathways during grain filling [72,73]. These contrasting expression patterns suggest a temporal shift in regulatory control from developmental to nutrient-responsive programs in the root system.

## 3. Discussion

Understanding the spatiotemporal coordination of nutrient transport is essential for improving nutrient use efficiency and grain quality in rice. In this study, we conducted a comprehensive transcriptome analysis covering key developmental stages and tissues, with a focus on nodes that function as central hubs for long-distance nutrient and assimilate allocation. Our time-course transcriptomic profiling of node I revealed distinct stage-specific gene expression programs across the reproductive phase. Particularly at the early grain filling stage, there was a marked upregulation of genes involved in mineral nutrient transport and allocation, carbohydrate transport, and metabolism. We observed that several mineral transporters were highly expressed in node I at either the early or late reproductive stages, reflecting their dynamic involvement in coordinating nutrient remobilization and acquisition. For example, *OsYSL2*, a metal-nicotianamine transporter mediating the long-distance transport of Fe and Mn from roots to grains, was strongly expressed during early stages (S1–S4), supporting its role in establishing reproductive tissue micronutrient supply [8]. *OsZIP3*, which unloads zinc from the xylem and redirects it toward the panicle via diffuse vascular bundles, also showed elevated expression during this period, facilitating Zn accumulation in developing grains [15]. As the plant transitioned into grain filling, several phosphate-related transporters became prominently upregulated in node I, including OsSULTR3;3, which mediates phosphate redistribution and also affects grain P and phytic acid content [27], and SPDT/OsSULTR3;4, whose loss-of-function reduces phosphorus accumulation in the grain by blocking phosphate transfer from node to panicle [4]. These findings reinforce the notion that node I dynamically adjusts transporter gene expression to support the nutrient needs of the developing grain.

In parallel, the efficient allocation of carbohydrate assimilates is equally critical for reproductive success. Among the sugar transporters identified, *OsSWEET13* and *OsSWEET14*, two clade III SWEET transporters responsible for sucrose efflux into phloem tissues and sink unloading, respectively, were significantly upregulated in node I at S5, consistent with their roles in facilitating carbon partitioning to the grain [28,29]. The coordinated expression of these transporters during early grain filling highlights a transcriptional program geared toward maximizing sugar delivery to sink organs. In addition to disaccharide export, monosaccharide transporters also contribute to intracellular sugar redistribution. OsMST6, a member of the monosaccharide transporter family, has been reported to mediate hexose uptake and redistribution in response to environmental signals [30]. Its elevated expression at S5 suggests a potential role in importing glucose or fructose into nodal cells, supporting local energy metabolism and possibly contributing to fine-tuned sugar flow during grain filling. Beyond sugar transport, enzymes involved in carbohydrate metabolism and remodeling also showed increased transcription at this stage. For instance, *OsPDT1*, encoding a galacturonosyl-transferase involved in pectin biosynthesis, was expressed at high levels during early reproductive development. While originally characterized for its role in tapetum development [32], its expression in node I may reflect a broader function in remodeling the nodal apoplast or vascular interface to facilitate efficient sugar unloading. Likewise, *MOC2*, which encodes a cytosolic fructose-1,6-bisphosphatase essential for sucrose biosynthesis, was upregulated during S5–S7. The moc2 mutant exhibits defects in sucrose accumulation and reduced yield [33], suggesting that MOC2 activity in node I supports sucrose regeneration or turnover during phloem unloading and redistribution.

Beyond temporal changes, spatial comparisons among tissues at the grain filling stage revealed that node I and node II harbor unique transcriptomic signatures compared to neighboring tissues such as the neck-panicle node, internode, and flag leaf. We identified hundreds of genes that were specifically expressed in node I or node II, including known transporters such as *OsVIT2*, *OsZIP3*, and *OsNR2*, and developmental regulators like *OsYAB4* and *PRL5*. GO and KEGG enrichment of these node-specific genes highlighted processes related to vascular morphogenesis, secondary metabolite biosynthesis, and signaling. Interestingly, many receptor-like kinases and protein kinases were also highly expressed in nodal tissues, implying that nodes are not only transport junctions but also signaling centers that integrate environmental and developmental cues to modulate nutrient allocation. The functional divergence between node I and node II suggests that these tissues coordinate complementary activities during grain filling: node I facilitates both nutrient influx from lower internodes and assimilate redirection from the flag leaf, whereas node II may regulate long-distance translocation and signaling.

Comparative transcriptomic analyses of the neck-panicle node between the booting and grain filling stages revealed a pronounced transcriptional reprogramming geared toward nutrient mobilization and assimilate unloading at grain filling stage. This developmental shift was marked by the strong induction of key transporters responsible for nitrogen, phosphorus, and sugar transport. For instance, *OsNRT1.1A* (also known as *OsNPF6.3*) and *OsNRT2.3*, two nitrate transporters with known roles in nitrate uptake and systemic nitrogen signaling, were significantly upregulated at the grain filling stage. OsNRT1.1A has been shown to enhance nitrogen use efficiency and grain yield by optimizing nitrate uptake and remobilization during reproductive development, particularly under fluctuating nitrogen availability [74]. OsNRT2.3, which produces two isoforms with distinct subcellular localization, mediates pH-dependent nitrate transport and plays a central role in nitrate allocation to sink tissues, including developing grains [68]. Their elevated expression in the NPN at grain filling suggests a critical function in delivering nitrate into the panicle to support rapid growth and protein biosynthesis during seed development. In addition to nitrate, the expression of multiple ammonium transporters, including *OsAMT1;1*, *OsAMT1;2*, *OsAMT2;1*, and *OsAMT3;2*, was also markedly increased at S5. These AMT family members facilitate high-affinity uptake and internal redistribution of ammonium, a major inorganic nitrogen source in rice cultivation systems. OsAMT1;1 and OsAMT1;2 are cooperatively required for the uptake of low level of ammonium in rice roots [75], while OsAMT2;1 and OsAMT3;2 are thought to mediate long-distance transport and tissue-specific remobilization of NH_4_^+^ [76,77]. Their upregulation in the NPN suggests that ammonium, along with nitrate, is actively delivered to the developing spikelets through this nodal checkpoint, contributing to nitrogen remobilization during early grain filling. Phosphorus-related transporters were also significantly upregulated in the NPN during S5. Notably, *OsPHT1;1*, *OsPHT1;8*, and *OsPHT4;1* were strongly expressed. These genes mediate phosphate uptake and intracellular translocation, and their expression aligns with the high phosphorus demand of developing reproductive tissues [78,79,80]. OsPHO1;1 and OsPHO1;3, which are involved in xylem loading and intervascular transfer of phosphate, were also induced, suggesting enhanced phloem-mediated P allocation to grains [81,82]. The increased expression of *OsSULTR3;3*, a member of the sulfate transporter family shown to facilitate radial phosphate movement [27], further supports a coordinated activation of a phosphate transport network at the NPN. The convergence of these P transporters indicates that the neck-panicle node plays a pivotal role in buffering and redirecting phosphorus fluxes from source organs into developing spikelets.

Comparison of node I and NPN transcriptomes at S5 enabled the identification of candidate genes potentially involved in nutrient distribution toward the flag leaf—a key trait distinguishing node I as a bidirectional allocation hub. For instance, *OsZIP3*, a node-localized zinc transporter responsible for unloading Zn from the xylem into adjacent vascular bundles [13], was more highly expressed in node I than in NPN, suggesting that node I may regulate Zn partitioning toward both flag leaves and panicles. Similarly, *OsLsi2*, a silicon efflux transporter, and *OsMOT1;2*, a molybdate transporter, were also enriched in node I. OsLsi2 is essential for Si transport toward the shoots and contributes to mechanical strength and stress resistance in reproductive tissues [3], while OsMOT1;2 facilitates Mo translocation to aerial organs, supporting molybdoenzyme activity required during grain development [83]. Their higher expression in node I points to specialized functions in upward nutrient routing not observed in the NPN. Together, these findings reveal a spatially resolved and developmentally coordinated gene expression program that fine-tunes the directionality and specificity of nutrient fluxes in rice nodes. While the NPN operates as a unidirectional conduit funneling nutrients and carbohydrates into the panicle, node I supports both upward and lateral redistribution, including toward the flag leaf. This distinction provides mechanistic insight into the division of labor among nodal tissues and offers molecular targets for manipulating nutrient partitioning to enhance grain nutrition and reduce the accumulation of undesirable elements such as Cd and As.

While efficient transport and redistribution of stored nutrients from leaves, stems, and nodes is essential for grain filling, sustained root absorption of mineral nutrients during this period is equally crucial to meet the elevated sink demand. Our results showed that a broad set of nutrient uptake genes was significantly upregulated in roots at the grain filling stage, reflecting an enhanced capacity for direct acquisition of essential elements during early grain filling. Among the phosphate transporters, *OsPHT1;4*, a member of the high-affinity phosphate transporter family, showed elevated expression at S5. This gene plays a central role in Pi acquisition under low-phosphate conditions, and its activation during grain filling suggests a compensatory mechanism to maintain phosphorus homeostasis as demand increases in reproductive organs [69]. For nitrogen uptake, both *OsNRT1.2* and *OsAMT1;3*, responsible for nitrate and ammonium uptake, respectively, were also induced at the grain filling stage. OsNRT1.2 functions in root-to-shoot nitrate loading into the xylem and has been implicated in regulating root architecture and nitrate signaling, while OsAMT1;3 supports ammonium acquisition, particularly under nitrogen-limited conditions [84]. Their coordinated upregulation ensures that nitrogen delivery to developing grains remains uninterrupted during peak demand. Similar strong upregulation of K and Si transporters were also observed. *OsHAK17*, a high-affinity potassium transporter involved in K^+^ uptake under a high-K environment [85], was upregulated during the grain filling stage, suggesting that sufficient K uptake is vital for maintaining turgor and cellular activity in developing sink tissues. *OsLsi1*, *OsLsi2*, and *OsLsi3*, which were responsible for silicon influx into root cells and its subsequent loading into the xylem, were all transcriptionally activated during grain filling. These transporters have been shown to increase grain yield and reduce lodging by promoting silicification in panicle and hull tissues, further emphasizing the importance of continued Si uptake during this stage [3,86].

*OsNRAMP5* and *OsCd1*, two divalent metal transporters, were upregulated at the grain filling stage. OsNRAMP5 is the primary route for Mn^2+^ and Cd^2+^ uptake from soil, while OsCd1 modulates Cd transport and its partitioning between roots and shoots. Notably, the high expression of both *OsNRAMP5* and *OsCd1* during grain filling coincides with enhanced root uptake and translocation of cadmium, which aligns with previous findings that grain Cd accumulation in rice primarily originates from root absorption rather than remobilization [87]. Their activation during S5 suggests an increased risk of Cd accumulation in grains at this stage, emphasizing the importance of tightly regulating these pathways to reduce Cd entry into the food chain. In addition to Cd, As is another toxic element of concern in rice. Silicon transporters such as OsLsi1, OsLsi2, and OsLsi3, while essential for Si uptake and beneficial for plant mechanical strength and stress resistance, are also known to facilitate arsenite uptake and translocation [88,89]. The observed upregulation of these genes at the grain filling stage, therefore, not only supports increased Si delivery to reproductive tissues but also suggests a potential concomitant enhancement in As accumulation in grains. This dual role highlights a trade-off between beneficial and harmful element transport during grain filling and reinforces the need for strategies that can selectively modulate transporter activity to favor nutrient enrichment while minimizing toxic metal accumulation.

In summary, our findings establish a comprehensive transcriptomic atlas of rice nodes and roots during reproductive growth and uncover stage- and tissue-specific gene expression programs involved in nutrient transport and allocation. This study identifies key transporters and regulatory genes that mediate the delivery of mineral elements and photoassimilates to developing grains. These insights provide a foundation for future functional studies and molecular breeding efforts aimed at improving nutrient use efficiency, grain nutritional value, and yield stability in rice and other cereal crops.

## 4. Materials and Methods

### 4.1. Plant Materials and Tissue Sampling

Rice plants (*Oryza sativa* L. ssp. japonica, cv. Nanjing 9108) were cultivated under standard field management conditions in a paddy field at the Baima experimental station of Nanjing Agricultural University (Nanjing, China). Tissue sampling was performed during the reproductive growth stage. The reproductive growth period was divided into seven representative developmental stages: early inflorescence development (inflorescence length ≈ 1 cm; S1), late inflorescence development (≈5 cm; S2), booting (≈25 cm; S3), heading (flowering; S4), grain filling (5 days after flowering, DAF; S5), late grain filling (15 DAF; S6), and seed maturation (25 DAF; S7). These stages were abbreviated as S1 to S7, respectively, throughout this study. Samples were collected at specific stages to capture temporal and spatial gene expression dynamics. At S1, root (RT) and node I (NDI, the highest node that connects the flag leaf) were collected; at S3, the neck-panicle node (NPN) and NDI were collected; and at S5, NPN, NDI, internode I (IN), node II (NDII), flag leaf blade (FLB), flag leaf sheath (FLS), and RT were sampled. At S2, S4, S6, and S7, only NDI was collected. To further probe spatial-functional divergence between nodal tissues, gene expression in NPN and NDI was compared at both S3 and S5, while root transcriptomes were analyzed at S1 and S5 to investigate dynamic shifts in nutrient acquisition capacity. All tissues were collected in three biological replicates, resulting in 15 tissues and 45 samples. For nodal tissues (NPN, NDI, and NDII), each biological replicate consisted of approximately 30 individual nodes. Immediately after collection, samples were frozen in liquid nitrogen and stored at −80 °C until RNA extraction and transcriptome analysis.

### 4.2. RNA Extraction, cDNA Library Construction, and Transcriptome Sequencing

Total RNA was extracted from each sample using the Plant Total RNA Extraction Kit (BioTeke, Beijing, China) according to the manufacturer’s instructions. The purity, concentration, and integrity of RNA were assessed using a NanoDrop spectrophotometer (Thermo Fisher Scientific, Waltham, MA, USA), Qubit 2.0 Fluorometer (Life Technologies, Carlsbad, CA, USA), and an Agilent 2100 Bioanalyzer (Agilent Technologies, Santa Clara, CA, USA), respectively. Only RNA samples meeting quality standards were used for subsequent library construction. For each qualified sample, mRNA was isolated using oligo(dT)-attached magnetic beads, followed by fragmentation in a fragmentation buffer. First-strand cDNA synthesis was performed using random hexamer primers and the fragmented mRNA as a template. Second-strand cDNA was then synthesized by adding PCR buffer, dNTPs, RNase H, and DNA polymerase I. End repair, A-tailing, and adapter ligation were subsequently performed. Fragments in the size range of 300–400 bp were selected using AMPure XP beads (Beckman Coulter, Brea, CA, USA). The adapter-ligated cDNA fragments were then amplified by PCR to generate the final cDNA library. The quality and concentration of the libraries were evaluated using Qubit 2.0, the Agilent 2100 Bioanalyzer, and quantitative PCR (qPCR). Libraries with concentrations >2 nM and appropriate insert sizes were considered qualified. All qualified libraries were pooled according to the required sequencing depth and sequenced using Illumina HiSeq2500 (Illumina, San Diego, CA, USA).

### 4.3. RNA-seq Data Processing

To obtain high-quality clean reads, raw reads were first filtered using fastp (version 0.18.0; https://github.com/OpenGene/fastp; accessed on 15 January 2023) with the following parameters: (1) remove reads containing adapters; (2) remove reads containing more than 10% unknown nucleotides (N); and (3) remove reads in which more than 50% of the bases had low quality scores (Q-value ≤ 10). The resulting paired-end clean reads were then mapped to the *Oryza sativa* reference genome (cv. Nipponbare, IRGSP-1.0; https://rapdb.dna.affrc.go.jp; accessed on 22 June 2022) using HISAT2 (version 2.2.4) with the parameter --rna-strandness RF and all other settings set to default. Insert size distribution and sequencing randomness were evaluated from the mapped data to assess library quality. For each sample, StringTie (version 1.3.1) was used to assemble mapped reads in a reference-guided manner and to optimize gene structure. Transcript abundance was quantified using the FPKM (Fragments Per Kilobase of transcript per Million mapped reads) method, which accounts for both transcript length and sequencing depth [11]. Expression values were calculated to evaluate transcriptional variation across samples and to support downstream analyses such as differential gene expression, alternative splicing, and novel transcript prediction.

### 4.4. Principal Component Analysis (PCA)

Principal Component Analysis (PCA) was used to assess the overall variation and relationships among transcriptome samples. As an unsupervised multivariate statistical method, PCA reduces high-dimensional gene expression data into a few orthogonal components (principal components) that capture the largest sources of variance. This facilitates intuitive visualization of sample clustering, detection of biological trends, and identification of potential outliers. PCA analysis was performed using the Metware Cloud platform (https://cloud.metware.cn) with default settings.

### 4.5. Short Time-Series Expression Miner (STEM) Analysis

To investigate temporal expression patterns of genes in node I across different reproductive growth stages, we conducted a time-series clustering analysis using Short Time-series Expression Miner (STEM) on the OmicShare platform (https://www.omicshare.com/tools, accessed on 11 September 2025). Normalized expression values were log2-transformed, and 20 distinct temporal profiles were predefined. Significant expression profiles were identified using a threshold of *p* < 0.05. Genes assigned to these significant profiles were extracted for further functional enrichment and regulatory network analysis.

### 4.6. Venn Diagram Analysis

To identify genes specifically upregulated in nodal tissues, we performed Venn diagram analysis using OECloud tools (https://cloud.oebiotech.com). Differentially expressed genes (DEGs) that were significantly upregulated in NDI and NDII compared to other tissues at the grain filling stage (S5) were selected, and the union of these gene sets was visualized and used for downstream functional analysis.

### 4.7. Identification of DEGs

To identify differentially expressed genes (DEGs), pairwise comparisons were performed between either different developmental stages or different tissue using the OECloud platform (https://cloud.oebiotech.com). Statistical significance of differential expression was assessed using the following criteria: false discovery rate (FDR) < 0.01, *p*-value < 0.05, and absolute fold change ≥ 2. Volcano plots were generated to visualize the overall DEG distribution. In addition, hierarchical clustering and heatmap visualization were performed using either OECloud tools or Genescloud (http://www.genescloud.cn), based on log2- or log10-transformed FPKM values.

### 4.8. GO and KEGG Analysis

To investigate the biological significance of DEGs, functional annotation and enrichment analyses were conducted based on the Gene Ontology (GO) and Kyoto Encyclopedia of Genes and Genomes (KEGG) databases. These analyses were performed using OECloud tools (https://cloud.oebiotech.com), with the organism set as *Oryza sativa* ssp. japonica and the database version set to PlantBiology v7.0. GO terms and KEGG pathways with *p* ≤ 0.05 were considered significantly enriched and were used to interpret the roles of DEGs in developmental processes, nutrient transport, and stress responses.

### 4.9. Validation Genes Specifically Expressed at S5 in Node I by qRT-PCR

To confirm the expression pattern of genes that are specifically highly expressed at S5 in node I, the relative expression level of 11 genes was determined by qRT-PCR. RNA was extracted from node I at S1–S7 by a Total Plant RNA Extraction Kit (BioTeke). A quantity of 1 μg of RNA was reverse-transcribed into cDNA using a HiScript II First-Strand cDNA Synthesis Kit (Vazyme, Nanjing, China). qRT-PCR was performed in a Real-Time PCR detection System (CFX96, Bio-Rad, Hercules, CA, USA) using 2× AceQ qPCR SYBR Green Master Mix (Vazyme). The program was 95 °C for 30 s, followed by 40 cycles of 95 °C for 10 s and 60 °C for 30 s. The relative gene expression level was presented as 2^−ΔCt^ with the rice *OsActin* gene as an internal control [90]. The primer sequences are listed in Table S12.

## 5. Conclusions

Based on the comprehensive transcriptomic analysis conducted in this study, we conclude that rice nodes, particularly node I, play a central and dynamic role in coordinating the distribution of mineral nutrients and carbohydrates during reproductive development. The temporal gene expression patterns revealed distinct stages of transporter gene activation, with a marked upregulation of key minerals (e.g., OsYSL2, OsZIP3, OsSULTR3;3, SPDT) and carbohydrate transporters (e.g., OsSWEET13, OsSWEET14, OsMST6) during the critical grain filling stage. Spatial comparisons identified tissue-specific regulatory networks involving nitrate and phosphate transporters in nodal and root tissues, emphasizing the specialized functions of the neck-panicle node in mobilizing nutrients to the panicle. Additionally, the root transcriptome highlighted the importance of ongoing nutrient uptake, including trace metal transporters (OsNRAMP5, OsCd1, OsLsi1, OsLsi2, OsLsi3), which contribute significantly to grain mineral accumulation, including potentially toxic elements. These findings establish a detailed spatiotemporal atlas of nutrient transporter gene activity in rice, enhancing our understanding of the molecular mechanisms underlying nutrient allocation and providing valuable targets for improving nutrient use efficiency and grain quality through genetic and breeding approaches.

## Data Availability

The RNA-seq datasets were deposited in Figshare (https://figshare.com (accessed on 1 August 2025)) with the DOI number: 10.6084/m9.figshare.30032290.

## Supplementary Materials

The following supporting information can be downloaded at: https://www.mdpi.com/article/10.3390/ijms26199357/s1.

## Abbreviations

The following abbreviations are used in this manuscript:

NDI	node I
NDII	node II
NPN	neck-panicle node
IN	internode I
FLB	flag leaf blade
FLS	flag leaf sheath
RT	root
