# Supplementary material for: Spatiotemporal Transcriptome Profiling Reveals Nutrient Transport Dynamics in Rice Nodes and Roots During Reproductive Development
Source: Int J Mol Sci. 2025 Sep 25;26(19):9357. doi: 10.3390/ijms26199357 (PMC12524880; doi:10.3390/ijms26199357)
Supplement: Supplementary file 1 [file ijms-26-09357-s001.zip › Node_transcriptome-SupplementlFigures-revised.pdf]

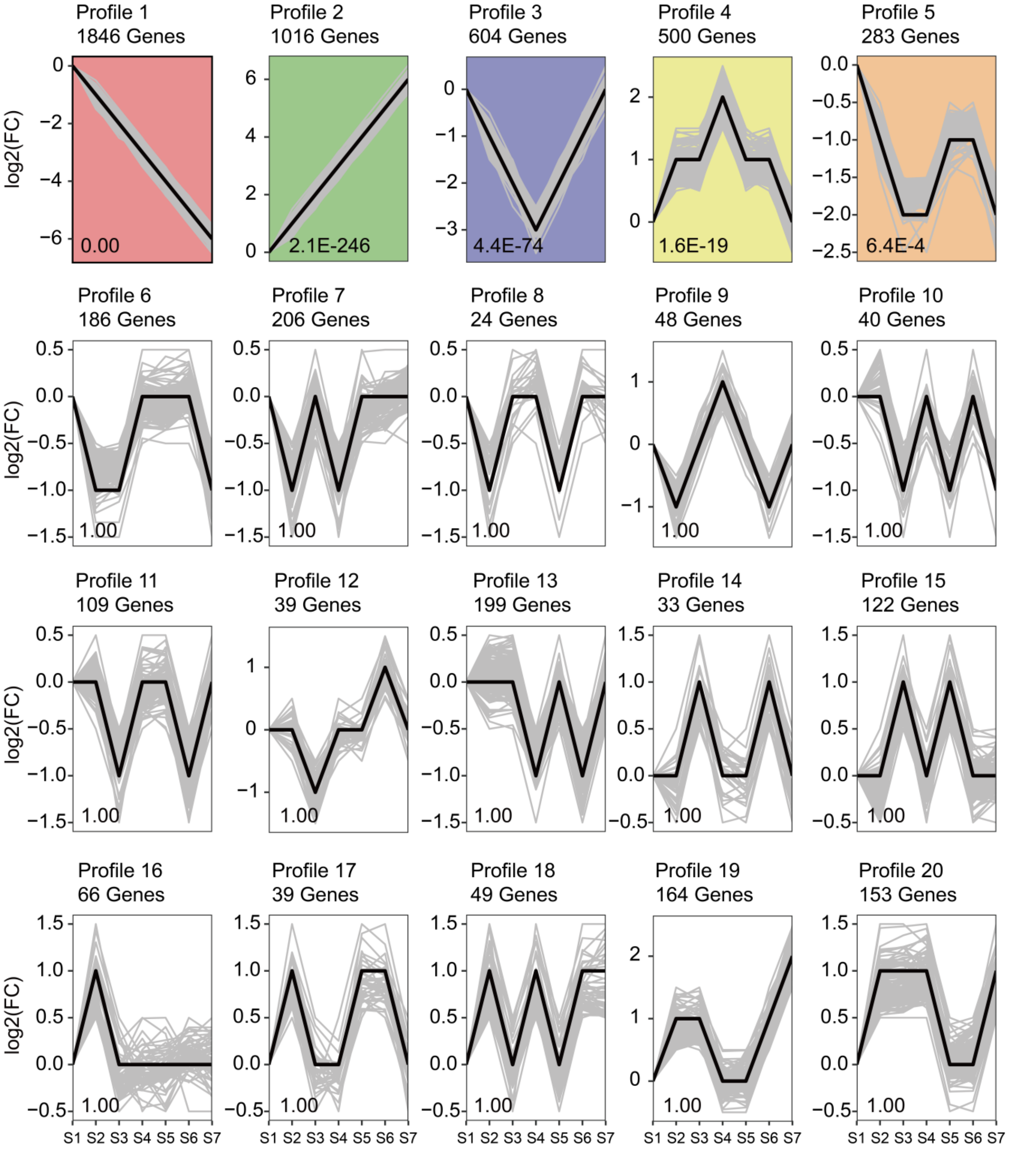

Supplemental figure S1. Short Time-series Expression Miner (STEM) analysis of genes in node I at seven reproductive growth stages. 20 profiles were identified and five significant profiles in color background were shown in Fig. 2. *p*-value were represented below each profile and gene numbers in each profile were showed on the top. Each gray line represents a gene, and the bold black line represents dynamic expression patterns.

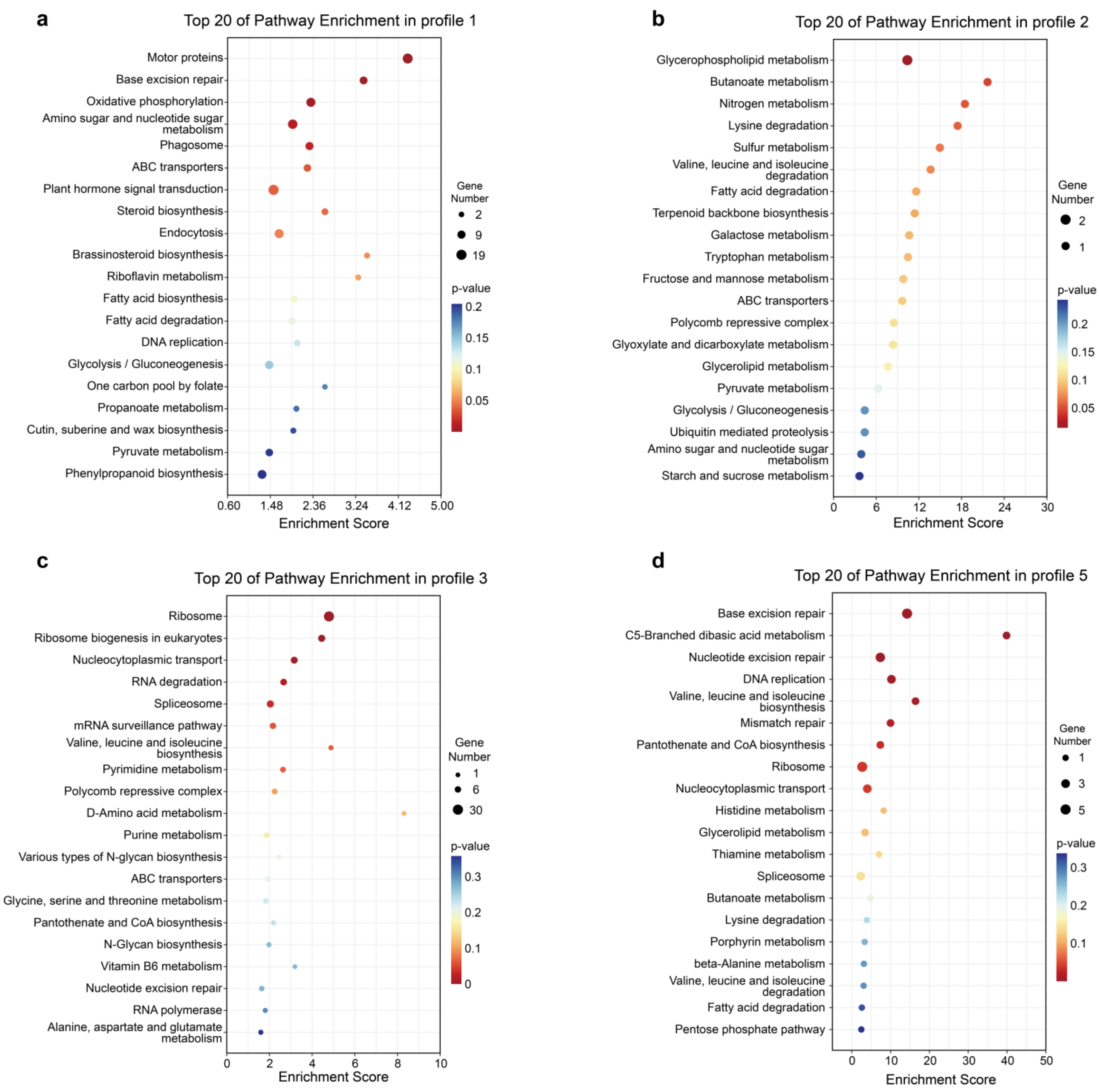

Supplemental figure S2. KEGG pathway enrichment analysis of genes in 4 profiles in Fig. 2. KEGG enrichment analysis of DEGs in the profile 1 (**a**), profile 2 (**b**), profile 3 (**c**), and profile 5 (**d**). The top 20 GO terms or KEGG pathway with lowest *p*-value were shown.

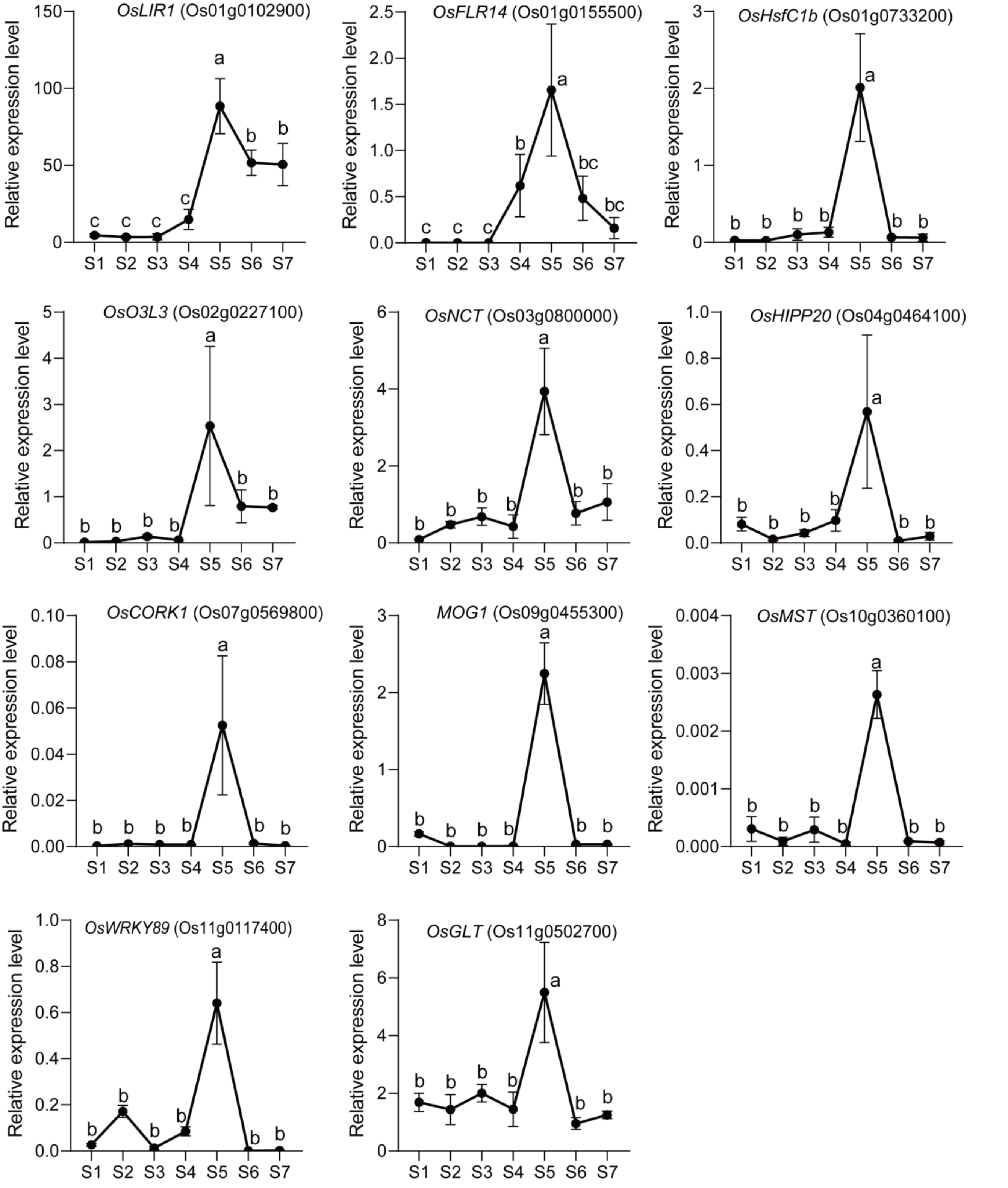

Supplemental figure S3. qRT-PCR Verification of the expression pattern of selected genes that are highly activated at the S5 stage in the node I. The relative expression levels were determined by qRT-PCR and presented as $2^{-\Delta Ct}$ using the rice *Actin* gene as internal reference gene. Different letters indicate significant differences of gene expression levels among different stage at $P \leq 0.05$ with three biological replicates (Tukey's multiple comparisons test).
